# Missed opportunities for venous thromboembolism prophylaxis during pregnancy and the postpartum period: evidence from mainland China in 2019

**DOI:** 10.1186/s12884-021-03863-w

**Published:** 2021-05-24

**Authors:** Zhekun Zhao, Qiongjie Zhou, Xiaotian Li

**Affiliations:** 1 Department of Obstetrics, Obstetrics and Gynecology Hospital of Fudan University, Shanghai, China; 2 Shanghai Key Laboratory of Female Reproductive Endocrine-Related Diseases, Shanghai, China; 3 Institute of Biomedical Sciences, Fudan University, Shanghai, China

**Keywords:** Venous thromboembolism, Prophylaxis, Pregnancy, Puerperium, China

## Abstract

**Background:**

Venous thromboembolism (VTE) has become one of the leading causes of maternal mortality. Thromboprophylaxis is recommended for the prevention of this condition; however, its use appears to be insufficient. Therefore, in this study, we aimed to identify the missed opportunities for VTE prophylaxis in hospitals that provide maternal healthcare in mainland China.

**Methods:**

In this cross-sectional survey-based study, we collected case data on pregnant and puerperal women with deep vein thrombosis and pulmonary thromboembolism from January 1st to December 31st, 2019. Demographics, obstetric information, VTE risk assessment scores, and prophylaxis-related information were recorded. Thromboprophylaxis included mobilization, mechanical methods, and treatment with anticoagulants.

**Results:**

Data corresponding to a total of 106 cases from 26 hospitals across China were collected, and 100 (94.3%) cases, 75 cases involving deep vein thrombosis and 25 cases involving pulmonary thromboembolism, were included in the final analysis. VTE occurred in 80% of the patients at the postpartum stage, while 20 patients developed the disease during the antenatal stage. Cesarean section, advanced maternal age, and obesity were the most common risk factors related to VTE during the postpartum stage, while a previous VTE-related history was a prominent risk factor among antenatal cases. Up to 75% of the patients had one or more missed opportunities for prophylaxis. The lack of the implementation of mechanical methods (60.8% vs. 24.5%, *P* < 0.001) and anticoagulant treatment (61.1% vs. 48.7%, P < 0.001) were more common in general hospitals compared to those of specialized hospitals. In women assessed as high-risk, anticoagulant treatment was lacking in 41 (54.7%) cases. More importantly, the lack of the implementation of mechanical methods was more common among women assessed as low-risk (56.0% vs. 38.7%, *P* < 0.001). Among the antenatal cases, the lack of treatment with anticoagulants (100.0% vs. 48.5%, *P* < 0.001) and implementation of mechanical methods (70.0% vs. 36.7%, *P* < 0.001) was highlighted. In addition, the lack of early mobilization was much more prominent among the PTE cases (10.5% vs. 37.5%, *P* < 0.001).

**Conclusions:**

At least one prophylactic opportunity was missed in most of the enrolled Chinese women with VTE during the course of their maternal healthcare. Missed prophylactic opportunities varied with the type of hospitals, risk assessment, onset timing and disease type. Further efforts from patients, healthcare systems, and healthcare providers are needed for improving the implementation of preventative strategies.

## Background

Venous thromboembolism (VTE), a condition that can present as either deep vein thrombosis (DVT) or pulmonary thromboembolism (PTE), has become a major health concern worldwide [1]. It is well recognized that pregnancy increases the risk of thromboembolism owing to conditions involving hypercoagulability, decreased mobility, and the compression of the inferior vena cava and pelvic veins [2–5]. In recent decades, VTE has become a leading cause of sudden death, accounting for 10–30% of all maternal deaths in the United States and other Western countries [6–8]. In China, the morbidity of obstetric VTE in 2012 was 50% higher than that in 2006 [9, 10]; moreover, the proportional ratio of maternal mortality has been found to increase yearly [11].

The rising incidence of VTE is possibly related to the increase in pregnancies among women of advanced age, more frequent use of assisted reproductive techniques, prevalence of metabolic diseases, reduction in mobilization, and ritual practice of “doing the month” (*Zuo yue zi*), which is the confinement and convalescence of Chinese women after childbirth [6, 12, 13]. In particular, with the rapid evolvement of the global coronavirus disease (COVID-19) pandemic, maternal infection and quarantine may potentially increase the maternal risk of VTE [7, 14, 15]. Therefore, VTE in pregnancy is an emerging health issue, and there is an urgent need for the implementation of appropriate prophylaxis. Prophylaxis for pregnancy-related VTE comprises of early mobilization, usage of mechanical methods (such as anti-embolism stockings (AES) and intermittent pneumatic compression (IPC) devices), and treatment with anticoagulants [2, 3, 16, 17]. These strategies have been recommended; however, the specific effects of their implementation are unclear. There is still a lack of related epidemiological evidence [18, 19].

In this study, we aimed to describe the thromboembolism cases and identify missed opportunities for thromboprophylaxis through the analysis of data collected via a case reporting survey that was conducted in 2019 across mainland China.

## Methods

### Study design

We collected data on pregnant and puerperal women with thromboembolisms from January 1st to December 31st, 2019, using a cross-sectional survey. All levels of hospitals that provide maternal healthcare services in mainland China were included in this VTE case reporting survey. Based on the Chinese National Network of Women’s and Children’s Health guidelines, a survey request was sent to the chief of the Department of Obstetrics at each hospital, and case report forms were completed and submitted by either the chief or senior physician at the participating hospitals. Hospitals that completed the survey and signed the informed consent document before February 29th, 2020, were enrolled for participation in the final analysis, and those with missing data on the survey were excluded. The institutional review board of the Obstetrics and Gynecology Hospital of Fudan University approved the project (IRB2020–147), and written informed consent was obtained from each participating hospital before enrollment.

### Data collection

All the cases involving patients who were diagnosed with DVT and/or PTE were considered eligible for the final analysis, and cases with missing information regarding the VTE diagnosis or risk scoring assessment were excluded. The VTE case reporting form consisted of the following four aspects:

1. General and clinical information regarding the patient with VTE: maternal age, height, weight, education level, residential area, parity, usage of artificial reproduction technique (ART), delivery mode, pregnancy complications including preterm birth, preeclampsia, postpartum hemorrhage, infection, and maternal comorbidities include cancer, heart disease, pulmonary disease, systemic lupus erythematosus, inflammatory bowel disease, gross varicose veins, diabetes mellitus, and sickle cell anemia.
2. Information regarding the VTE diagnosis: hospital type (general hospital or specialized hospital), diagnostic method (D-dimer test, ultrasound of the lower extremity vein, and computed tomography pulmonary angiography (CTPA)), disease onset (antenatal, intrapartum, or postpartum), and disease type (DVT or PTE). Specialized hospital refers to obstetrics hospitals, and generals hospitals refers to those non-specialized hospitals treating patients suffering from varied medical conditions.
3. Risk assessment score: a risk assessment scale that was designed according to the Royal College of Obstetricians and Gynecologists (RCOG) guidelines was used to determine the score. Accordingly, 1 point indicated age > 35 years, parity ≥2, use of assisted reproductive technology, multiple pregnancies, elective Cesarean section, body mass index (BMI) ≥ 30 kg/m^2^, antenatal smoking > 10 cigarettes per day, preeclampsia, postpartum hemorrhage (≥ 1000 ml or blood transfusion), gross varicose veins, preterm birth, stillbirth, antenatal immobility (≥ 7 days bed rest), ante- or postnatal infection, and prolonged labor (≥ 24 h); 2 points indicated emergency Cesarean section and BMI ≥ 40 kg/m^2^; 3 points indicated maternal comorbidities (cancer, heart disease, pulmonary disease, systemic lupus erythematosus, inflammatory bowel disease, diabetes mellitus, and sickle cell anemia), previous occurrence of VTE after major surgery, and known high-risk of thrombophilia; and 4 points indicated previous occurrence of VTE, apart from a single VTE event related to major surgery. Women with a total antenatal score of ≥3 or postnatal score of ≥2 were classified as high-risk, and the others were classified as low-risk.
4. Prophylaxis management strategies: VTE prophylaxis was based on published guidelines [2, 3]. VTE prophylaxis included early mobilization (such as off-bed activity or walking in the corridor within 24 h after delivery), use of mechanical modalities (use of AES or IPC), and treatment with anticoagulants. Early mobilization referred to postpartum mobilization.

### Statistical analysis

We used numbers and percentages to describe the characteristics of the patients in the reported VTE cases and those with missed prophylactic opportunities. The differences in the characteristics were examined by performing χ^2^ tests. Statistical analyses were performed using SPSS version 26.0 (IBM Corp., Armonk, N.Y., USA).

## Results

The data of a total of 106 cases from 26 hospitals were collected through a case reporting survey conducted in mainland China from January 1st to December 31st, 2019. After excluding cases with missing information regarding the VTE diagnosis, a total of 100 (94.3%) cases were finally included in the analysis.

In general, 74% of the patients were of maternal age > 35 years, and 76% had BMI < 30 kg/m^2^. Further, 68% of the patients were from eastern China, and 21 and 10% were from central and western China, respectively. Nearly half of the women were undergraduates or had a higher education level (47%), and 80% of the women underwent a Cesarean section. Regarding disease characteristics, 75% of the women were at a high risk for VTE, whereas the remaining 25% were at a low risk. In 80% of VTE cases the disease occurred in the postpartum period, while in 20% of VTE cases the disease occurred in the antenatal period. In addition, 76 patients had DVT, while 24 patients had PTE (Table 1). This national pattern was reflected both in the general hospital and specialized hospital subgroups. VTE cases in the general hospitals were mostly from the eastern region (84.3%), while 51.0% of the cases in the specialized hospitals were from the eastern region.

**Table 1:** Demographic and clinical characteristics of pregnant and puerperal women with venous thromboembolism by hospital type

		Total	General hospital	Specialized hospital	*P* value
**Age, years**	≤ 35	74 (74.0)	38 (74.5)	36 (73.5)	1.000
	> 35	26 (26.0)	13 (25.5)	13 (26.5)	
**BMI, kg/m**^**2**^	< 30	76 (76.0)	39 (76.5)	37 (75.5)	1.000
	≥ 30	24 (24.0)	12 (23.5)	12 (24.5)	
**Region of residence**^**a**^	Eastern region	68 (68.0)	43 (84.3)	25 (51.0)	0.001
	Central region	21 (21.0)	4 (7.8)	17 (34.7)	
	Western region	11 (11.0)	4 (7.8)	7 (14.3)	
**Education level**	High school or lower	24 (24.0)	14 (27.5)	10 (20.4)	0.133
	Undergraduate or higher	47 (47.0)	19 (37.5)	28 (57.1)	
	Missed	29 (29.0)	18 (35.3)	11 (22.5)	
**Delivery mode**	Vaginal delivery	20 (20.0)	12 (23.5)	8 (16.3)	0.646
	Cesarean section	80 (80.0)	39 (76.5)	41 (83.7)	
**Risk assessment**	Low risk	25 (25.0)	15 (29.4)	10 (20.4)	0.359
	High risk	75 (75.0)	36 (70.6)	39 (79.6)	
**Onset timing**	Antepartum	20 (20.0)	17 (33.3)	3 (6.1)	0.002
	Postpartum	80 (80.0)	34 (66.7)	46 (93.9)	
**Disease type**	DVT	76 (76.0)	38 (74.5)	38 (77.6)	0.899
	PTE	24 (24.0)	13 (25.4)	11 (22.4)	
**Total**		**100**	**51**	**49**	

Data are presented as number (percentage)

*VTE* Venous thromboembolism, *BMI* Body mass index, *DVT* Deep venous thrombosis, *PTE* Pulmonary thromboembolism

^a^Region of residence. *Eastern region*: Beijing, Fujian, Guangdong, Jiangsu, Liaoning, Shandong, Shanghai, Tianjin, and Zhejiang. *Central region*: Anhui, Hainan, Hebei, Heilongjiang, Henan, Hubei, Hunan, Jiangxi, Jilin, and Shanxi. *Western region*: Chongqing, Gansu, Guangxi, Guizhou, Inner Mongolia, Ningxia, Qinghai, Shaanxi, Sichuan, Tibet, Yunnan, and Xinjiang

The main relevant risk factors are listed in Table 2. Among all the patients, Cesarean section, advanced maternal age, and obesity were the most common risk factors. A similar pattern was reflected in the postpartum subgroup. Moreover, a previous history of VTE, maternal obesity, and advanced age accounted for the predominant risk factors in nearly 80% of the antenatal cases.

**Table 2:** Main risk factors in pregnant and puerperal patients with antenatal and postpartum venous thromboembolism

Antenatal VTE (***N*** = 20)	Postpartum VTE (***N*** = 80)
Previous VTE history:5 (25.0)	Elective CS: 33 (41.8)
BMI ≥ 30 kg/m^2^: 5 (25.0)	Emergency CS: 30 (38.0)
Age > 35 years old: 4 (20.0)	Age > 35 years old: 21 (26.6)
Maternal comorbidities^a^: 3 (15.0)	BMI ≥ 30 kg/m^2^: 19 (24.1)
ART: 3 (15.0)	Maternal comorbidities^a^: 14 (17.7)
Parity > 2: 3 (15.0)	Parity > 2: 14 (17.7)
Multiple pregnancy: 3 (15.0)	Preterm birth: 12 (15.2)
Immobility (≥ 7 days bed rest): 3 (15.0)	Preeclampsia: 11 (13.9)
Previous thrombophilia: 2 (10.0)	PPH (≥ 1000 ml or blood transfusion): 11 (13.9)
	Infection: 7 (8.9)

Data are presented as number (percentage)

*VTE* Venous thromboembolism, *BMI* Body mass index, *CS* Cesarean section, *PPH* Postpartum hemorrhage, *ART* Assisted reproductive technology

^a^Maternal comorbidities include cancer, heart disease, pulmonary disease, systemic lupus erythematosus, inflammatory bowel disease, gross varicose veins, diabetes mellitus, and sickle cell anemia

Missed prophylactic opportunities among enrolled women are demonstrated in Table 3. It was found that one or more opportunities for VTE prophylaxis were missed in 75% of the total number of cases. The lack of the implementation of mechanical methods (60.8% vs. 24.5%, *P* < 0.001) and anticoagulant treatment (61.1% vs. 48.7%, *P* < 0.001) were more common in general hospitals compared to those of specialized hospitals. In women assessed as high-risk, anticoagulant treatment was lacking in 41 (54.7%) cases. More importantly, the lack of the implementation of mechanical methods was more common among women assessed as low-risk (56.0% vs. 38.7%, *P* < 0.001). Among the antenatal cases, the lack of treatment with anticoagulants (100.0% vs. 48.5%, *P* < 0.001) and implementation of mechanical methods (70.0% vs. 36.7%, *P* < 0.001) was highlighted. In addition, the lack of early mobilization was much more prominent among the PTE cases (10.5% vs. 37.5%, *P* < 0.001).

**Table 3:** Missed prophylactic opportunities among pregnant and puerperal women with venous thromboembolism

Prophylaxis	Hospital type		Risk assessment^**a**^		Onset timing		Disease type	
	General ***N*** = 51	Specialized ***N*** = 49	Low risk ***N*** = 25	High risk ***N*** = 75	Antenatal ***N*** = 20	Postpartum ***N*** = 80	DVT ***N*** = 76	PTE ***N*** = 24
**Missed prophylactic opportunities**^**b**^								
No early mobilization^**c**^	10 (19.6)	7 (14.3)	5 (20.0)	12 (16.0)	–	11 (13.9)	8 (10.5)	9 (37.5)
No use of mechanical methods	31 (60.8)	12 (24.5)	14 (56.0)	29 (38.7)	14 (70.0)	29 (36.7)	29 (38.2)	14 (58.3)
No use of anticoagulants^**d**^	22/36 (61.1)	19/39 (48.7)	–	41 (54.7)	9/9 (100)	32/66 (48.5)	35/56 (62.5)	6/19 (31.6)
**Numbers of missed prophylactic opportunities**^**d**^								
No opportunity missed	11 (21.6)	14 (28.6)	4 (16.0)	21 (28.0)	0 (0.0)	24 (30.4)	6 (7.9)	6 (25.0)
1 opportunity missed	12 (23.5)	29 (59.2)	8 (32.0)	33 (44.0)	6 (30.0)	35 (44.3)	19 (25.0)	3 (12.5)
2 opportunities missed	20 (39.2)	2 (4.1)	13 (52.0)	14 (18.7)	14 (70.0)	14 (17.7)	32 (42.1)	9 (37.5)
3 opportunities missed	8 (15.7)	4 (8.2)	–	7 (9.3)	–	6 (7.6)	19 (25.0)	6 (25.0)

Data are presented as number (percentage)

^a^Patients with a total score ≥ 3 before delivery or ≥ 2 after delivery were considered as high-risk patients, and the others, as low-risk patients

^b^Missed prophylaxis opportunities for postnatal patients include no early mobilization, no use of any mechanical methods, and no use of anticoagulants, while for antenatal patients they include no use of any mechanical methods and no use of anticoagulants since antenatal patients always practiced mobilization

^c^Early mobilization refers to mobilization after delivery

^d^The percentage of “no use of anticoagulants” refers to women who did not take anticoagulants and were stratified as high risk

## Discussion

In this cross-sectional survey-based study of 100 cases involving the development of VTE during pregnancy or the postpartum period, which were reported on by 26 hospitals across mainland China, we found that the use of prophylaxis is insufficient in pregnant and puerperal women. Up to 75% of the analyzed cases involved missed opportunities for prophylaxis during the course of the local maternal healthcare. The implementation of mechanical methods and treatment with anticoagulants were the most commonly missed opportunities among the cases in general hospitals or in antenatal period. Compared with in women assessed as high-risk or those in the postpartum period, missed opportunities by implementing of mechanical methods were more prevalent in women assessed as low risk; compared to DVT cases, the lack of early mobilization was much more prominent among the PTE cases. Therefore, implementing and improving prophylaxis through measures taken by patients, healthcare systems, or healthcare providers are required to consider variation of the type of hospitals, risk assessment, onset timing and disease type to halt the increasing risk of VTE.

The main strength of this study was its coverage of different hospitals and regions across mainland China, which were included in the analysis of risk assessment scores, disease onset, and disease type. The survey conducted was based on the case reporting form filled in by obstetric chiefs or experienced physicians. Due to the ethical concerns associated with a prospectively designed study on VTE, case reporting is invaluable, as it provides the data required to perform a VTE prophylaxis analysis for investigating prophylaxis implementation.

First, our study uncovered that approximately 75% of the 100 VTE cases involved one or more missed prophylaxis opportunities, indicating that prophylaxis was not well implemented, especially in terms of the use of mechanical methods and anticoagulants. It is known that thromboprophylaxis greatly reduces VTE-related maternal morbidity although it cannot prevent each onset of VTE. The missed chances for thromboprophylaxis have not been reported and analyzed yet among Chinese women during pregnancy and the puerperium period. A few relevant studies were conducted in developed countries, such as the United States and France, and in developing countries, such as those in Africa, Europe, the Middle East, and South Asia [20–24]. It was found that the physicians’ level of awareness of VTE prophylaxis was relatively high, but their decisions regarding the use of pharmacological thromboprophylaxis or mechanical thromboprophylaxis greatly varied [22, 23]. Since prophylaxis is the key factor that could aid in reducing VTE-related maternal mortality and morbidity through timely diagnosis and multidisciplinary treatment modalities, it is necessary to focus on the sufficiency of prophylaxis. This maybe related to resource availability, effective guidelines, multidisciplinary implementation, and patient compliance. Thus, further education is required not only for physicians but also for patients in order to increase the awareness of disease severity and early identification.

This study also revealed a tendency for missed opportunities for sufficient prophylaxis in antepartum women or low-risk women. Regarding antenatal patients, the evidence suggests that clinicians find the existing risk assessment method difficult to apply in practice [25]. It has been a consistent finding that there is a tendency to make poorer efforts toward prophylaxis in the case of low-risk pregnant women [26] possibly due to the underestimation of the possibility of the development of VTE. We propose that implementation of VTE prophylaxis should be a crucial step in maternal healthcare not only for high-risk women but also for low-risk women. Moreover, this preventive strategy is also required during pregnancy rather than only during the postpartum period.

Our study has several limitations. First, the hospitals that participated in the case reporting survey were tertiary and secondary hospitals, and no primary hospitals were enrolled. Further investigation is needed to evaluate the thromboprophylactic measures in primary hospitals, considering that the severity of the patients’ condition, qualification of physicians, and availability of medical equipment differ from those in secondary and tertiary hospitals. Second, this survey was based on a questionnaire; therefore, the presence of a potential reporting bias should be considered. The chief or senior physicians from the obstetrics department completed the survey, and trained staff members were assigned for data quality control. The point of contact could be contacted by telephone in case of any queries, which ensured the quality of the survey results to some extent.

## Conclusions

In summary, in the local health care setting in China, at least one prophylactic opportunity was missed in most of the women with VTE that developed during the antenatal or postpartum period. Missed prophylactic opportunities varied with the type of hospitals, risk assessment, onset timing and disease type. Further efforts are needed to improve the implementation of prophylaxis in women during pregnancy and the puerperium period, and such strategies may have the potential to be adopted in other countries around the world.

## Data Availability

The datasets obtained and/or analyzed during the current study are available from the corresponding author on reasonable request.

## Abbreviations

VTE: Venous thromboembolism
DVT: Deep venous thrombosis
PTE: Pulmonary thromboembolism
BMI: Body mass index
AES: Anti-embolism stockings
IPC: Intermittent pneumatic compression
